# A Preoperative Nomogram for the Prediction of High-Volume Central Lymph Node Metastasis in Papillary Thyroid Carcinoma

**DOI:** 10.3389/fendo.2021.753678

**Published:** 2021-12-22

**Authors:** Peiliang Lin, Faya Liang, Jingliang Ruan, Ping Han, Jianwei Liao, Renhui Chen, Baoming Luo, Nengtai Ouyang, Xiaoming Huang

**Affiliations:** ^1^ Guangdong Provincial Key Laboratory of Malignant Tumor Epigenetics and Gene Regulation, Sun Yat-sen Memorial Hospital, Sun Yat-sen University, Guangzhou, China; ^2^ Department of Otolaryngology, Sun Yat-sen Memorial Hospital, Sun Yat-sen University, Guangzhou, China; ^3^ Department of Ultrasound, Sun Yat-sen Memorial Hospital, Sun Yat-sen University, Guangzhou, China; ^4^ Cellular and Molecular Diagnostics Center, Sun Yat-sen Memorial Hospital, Sun Yat-sen University, Guangzhou, China

**Keywords:** thyroid papillary carcinoma, lymphatic metastasis, neck, nomograms, reoperation

## Abstract

**Background:**

High-volume lymph node metastasis (HVLNM, equal to or more than 5 lymph nodes) is one of the adverse features indicating high recurrence risk in papillary thyroid carcinoma (PTC) and is recommended as one of the indications of completion thyroidectomy for patients undergoing thyroid lobectomy at first. In this study, we aim to develop a preoperative nomogram for the prediction of HVLNMs in the central compartment in PTC (cT_1-2_N_0_M_0_), where preoperative imaging techniques perform poor.

**Methods:**

From October 2016 to April 2021, 423 patients were included, who were diagnosed as PTC (cT_1-2_N_0_M_0_) and underwent total thyroidectomy and prophylactic central compartment neck dissection in our center. Demographic and clinicopathological features were recorded and analyzed using univariate and multivariate logistic regression analysis. A nomogram was developed based on multivariate logistic regression analysis.

**Results:**

Among the included patients, 13.4% (57 cases) were found to have HVLNMs in the central compartment. Univariate and multivariate logistic regression analysis showed that age (</=35 years vs. >35 years), *BRAF* with V600E mutated, nodule diameter, and calcification independently predicted HVLNMs in the central compartment. The nomogram showed good discrimination with an AUC of 0.821 (95% CI, 0.768–0.875).

**Conclusion:**

The preoperative nomogram can be used to quantify the probability of HVLNMs in the central compartment and may reduce the reoperation rate after thyroid lobectomy.

## Introduction

As several studies indicated that oncologic outcomes of lobectomy are comparable to those of a near-total or total thyroidectomy for low-risk papillary thyroid carcinoma (PTC) (1–3), thyroid lobectomy alone is recommended as an alternative for low-risk PTC (4–6). However, some adverse pathological features, which would upgrade the cases from low risk to intermediate risk or high risk, could only be assessed in postoperative examination (7, 8). Some of these adverse pathological features are recommended as indications of completion thyroidectomy following lobectomy, which may lead to more severe anxiety for the patients and increase expenditures for medical care and public health, compared to one-stage near-total or total thyroidectomy (7, 9). In NCCN clinical practice guidelines in oncology (thyroid carcinoma, version 3.2021), large volume pathological N1a metastasis is recommended as one of the adverse pathological features indicating completion thyroidectomy following lobectomy. It refers to equal to or more than 5 involved lymph nodes or any nodal metastasis >2 mm in largest dimension, which reveals high risk of PTC recurrence (7, 10, 11), whereas the diagnostic performance of imaging techniques, such as ultrasonography (US) or computer tomography, is poor in detecting involved lymph nodes in the central compartment preoperatively (12). In this research, we tried to build a preoperative nomogram to predict high-volume central lymph node metastasis (HVCLNMs, equal to or more than 5 involved lymph nodes in central compartment) to reduce reoperation rate after thyroid lobectomy.

## Materials and Methods

### Patients

From October 2016 to April 2021, a total of 423 patients undergoing total thyroidectomy and prophylactic central compartment neck dissection in our center were included. Preoperative diagnoses were revealed by US. The most suspicious malignant thyroid nodule with KWAK-TIRADS of 4 to 5 in either thyroid lobe in preoperative US examination was confirmed by means of ultrasound-guided fine-needle aspiration cytologic examination (FNAC) (13).

The eligibility criteria were as follows: (1) unilateral papillary thyroid carcinoma diagnosed in preoperative US and FNAC (2); the largest malignant nodule size </= 4.0 cm in preoperative US examination; and (3) undergoing total thyroidectomy and prophylactic central compartment neck dissection in our center.

The exclusion criteria were (1) bilateral malignant thyroid nodules diagnosed in FNAC; (2) any nodule of contralateral thyroid lobe with KWAK-TIRADS of 4c or 5, even without positive FNAC results; (3) malignant or suspicious malignant thyroid nodules located on isthmus of thyroid gland either with positive FNAC results or with KWAK-TIRADS of 4c or 5 in US; (4) extrathyroidal extension; (5) enlarged lymph nodes on palpation or US scan; (6) clinical evidence of distant metastases such as in the lung and bone; and (7) history of previous neck radiation exposure.

For patients who met all the inclusion criteria but none of the exclusion criteria, the advantages and disadvantages of the two operation procedures, total thyroidectomy and thyroid lobectomy, were explained, and the patients chose either procedure based on preference. Those choosing total thyroidectomy were included in our study.

Demographic features, the *BRAF V600E* mutation test result, serum thyroperoxidase antibody (TPOAb) and thyroglobulin antibody (TgAb) levels, US, FNAC, and pathological examination results were recorded and analyzed. Hashimoto’s thyroiditis was diagnosed by demonstration of elevated circulating autoantibodies to thyroid antigens and reduced echogenicity on thyroid sonogram (14, 15). In our research, the size of metastatic foci was not taken into consideration in predicting HVCLNMs, as lymph node could be cut perpendicularly, parallelly, or at any angle to the longest dimension of metastatic foci and the diameter of metastatic foci in largest dimension is difficult to obtain actually.

The study was carried out with approval of the Institutional Review Board (IRB) of our hospital (approval No. SYSEC-KY-KS-2020-149).

### Ultrasound Characteristics

The ultrasound imaging characteristics of the largest malignant nodule were assessed by two researchers (with 5–10 years of experience), independently. The imaging characteristics included the composition, size, shape (the anteroposterior dimension divided by its transverse dimension, A/T), echogenicity, margins, calcification, and vascularity pattern. Nodule composition was classified as solid or mixed composition. Nodule size refers to the maximum diameter. Shape was classified as A/T </= 1 or A/T > 1. The echogenicity was categorized as hyper-, iso-, hypoechogenicity, or marked hypoechogenicity. Margins were classified as well-circumscribed, irregular, or microlobulated, and the calcification pattern was categorized as noncalcification, microcalcification, macrocalcification, or peripheral (rim) calcification. Microcalcifications were defined as hyperechoic foci that were equal to or less than 1 mm in diameter. When calcifications were larger than 1 mm, they were classified as macrocalcifications. When microcalcifications presented in a nodule, regardless of whether macrocalcifications and/or peripheral (rim) calcifications existed at the same time, they were classified as microcalcifications. The color Doppler flow pattern of a lesion is classified into four types: (1) perinodular vascularity, (2) intranodular vascularity, (3) mixed perinodular and intranodular vascularity, and (4) absence of blood flow. Masses with mixed components were evaluated based on the internal solid components. Whether malignant nodule (diagnosed in FNAC examination) or suspicious malignant nodule (classified as KWAK-TIRADS 4c or 5 in US, but without positive FNAC results) is located at the inferior part (lower third) or superior part (upper third) of the thyroid lobe was also recorded. Discrepancies between the two researchers were resolved by rechecking the images and discussing with another researcher (with more than 20 years of experience).

### Identification of *BRAF V600E* Mutations

The results of *BRAF V600E* mutation test using preoperative qPCR were recorded.

In preoperative qPCR, DNA extraction was performed on fine-needle cytologic samples using the AmoyDx^®^ FFPE DNA Kit (Amoy Diagnostics Co., Ltd., Xiamen, China) according to the manufacturer’s protocols. The concentration and purity of extracted DNA were assessed by Nanodrop spectrophotometry.

*BRAF V600E* mutation detection was performed in the Cellular and Molecular Diagnostic Centre of Sun Yan-Sen Memorial Hospital, Sun Yat-Sen University. DNA from the 423 patients was tested using the AmoyDx^®^ *BRAF* Mutations Detection Kit (Amoy Diagnostics Co., Ltd., Xiamen, China) under the principle of the amplification refractory mutation system (ARMS), detecting *BRAF V600E* mutation (exon 15). AmoyDx^®^ *BRAF* Mutations Detection Kit was used to detect *BRAF V600E* mutation in cytological specimens and tissue specimens in some previous studies (16, 17). Briefly, the PCR was carried out on a 7500 Real‐Time PCR System (Applied Biosystems) according to the manufacturer’s protocol with 10 ng of DNA in each reaction system. The PCR kit allows an LOD (limit of detection) as low as 1% for *BRAF V600E* (PCR kit instructions). All results were confirmed according to the criterion suggested by the manufacturer.

### Statistical Analysis

Qualitative variables were summarized as absolute and relative frequency (percentage). Quantitative variables were summarized as the means and standard deviations (mean ± SD) whenever data proved to be normally distributed; otherwise, the medians and interquartile ranges were used (interquartile range is provided in parentheses within the manuscript).

Logistic regression was used to model the association between each variable and the presence of high-volume lymph node metastases (equal to or more than 5 involved lymph nodes) in the central compartment. Variables with a univariate *p*-value less than 0.1 were included in multivariate analysis. The multivariable logistic regression model was calculated using likelihood ratio statistics to identify significant and independent variables with forward stepwise model. Thresholds to determine whether variables were enrolled in the model was set as *p* < 0.05 for variables entering the model, and *p* > 0.10 for variables rejected, which were set to guard against the possibility of having the program enter and remove the same variable at successive steps (18).

To provide surgeons with a quantitative tool to predict the individual probability of high-volume lymph node metastasis (HVLNM) in the central compartment, we built the diagnostic nomogram using the independent predictors selected by multivariable logistic regression analysis to generate a combined indicator. The ability of the model to discriminate between patients with and without HVCLNMs was assessed using the area under the ROC curve (AUC), also known as the concordance index, whose value ranged from 0.50 to 1.00. A value of 1.00 indicated perfect discrimination, while a value of 0.50 indicated random predictions.

One thousand random bootstrap resamples were used for internal validation of the model to address model overfit and obtain a relatively unbiased evaluation. Calibration curves were plotted to assess the calibration of the diagnostic nomogram, which was assessed by plotting the predicted versus the actual probability of HVCLNMs. The absolute error of the nomogram prediction was measured by the distance between the pairs and the 45° line (19).

All statistical analyses were performed in the rms/pROC package in R version 4.0.0 and SPSS software (version 21.0, Chicago, IL, United States). A *p*-value of < 0.05 was considered to indicate statistical significance.

## Results

In our study, a total of 423 patients with age of 47.0 ± 12.2 years were included. Among the patients, 77 (18.2%) were male. The *BRAF* mutation test results showed that 372 (87.9%) had a V600E mutation. A total of 112 (26.5%) patients were diagnosed as Hashimoto’s thyroiditis preoperatively according to circulating autoantibodies and ultrasonic features. The characteristics of the thyroid nodules in ultrasound examination and postoperative pathological features are presented in **Table 1**. The intra-observer agreements of the two researchers in assessing US characteristics were 0.773–0.966 and 0.839–0.977, respectively, whereas the inter-observer agreements between the two researchers were 0.759–0.947 (**Table S1**).

**Table 1:** Clinicopathological characteristics of the included patients.

Characteristics	Value
No. of patients	423
Gender	
Male	77 (18.2%)
Female	346 (81.8%)
Age (years)	47.0 ± 12.2
</=35 years	85 (20.1%)
>35 years	338 (79.9%)
BRAF with V600E mutated	372 (87.9%)
Hashimoto’s thyroiditis	112 (26.5%)
US Characteristics	
Multifocality	
No focus with TIRADS of 4c/5	224 (53.0%)
Single focus with TIRADS of 4c/5	94 (22.2%)
Multiple Foci with TIRADS of 4c/5	105 (24.8%)
TIRADS of 4a/4b/4c/5	31/78/49/265
Diameter/mm	9.8 (6.5, 15.5)
Composition	
Mixed composition	8 (1.9%)
Solid composition	415 (98.1%)
Shape	
A/T > 1	157 (37.1%)
A/T </= 1	266 (62.9%)
Echogenicity	
Hyperechogenicity	3 (0.7%)
Isoechogenicity	114 (27.0%)
Hypoechogenicity	274 (64.8%)
Marked hypoechogenicity	32 (7.6%)
Margin	
Well-circumscribed Margin	202 (47.8%)
Microlobulated Margin	212 (50.1%)
Irregular Margin	9 (2.1%)
Calcification	
No Calcifications	153 (36.2%)
Macrocalcifications	202 (47.8%)
Microcalcifications	60 (14.2%)
Rim Calcification	8 (1.9%)
Vascularity Pattern	
Absence	252 (59.6%)
Perinodular	37 (8.7%)
Intranodular	127 (30.0%)
Perinodular and intranodular	7 (1.7%)
SMF at inferior part of thyroid lobe	56 (13.2%)
SMF at superior part of thyroid lobe	90 (21.3%)
Pathological Features	
T status of T1a/T1b/T2	259/114/50
Diameter/mm	9 (6, 14)
Multifocality	255 (60.3%)
N status of N0/N1a	238/185
Dissected Lymph Nodes	5 (2, 9)
Positive Lymph Nodes	0 (0, 2)
HVCLNMs/cases	57 (13.4%)

A/T, the anteroposterior dimension divided by its transverse dimension; HVCLNMs, high-volume central lymph node metastasis (equal to or more than 5 lymph nodes); SMF, suspicious malignant foci; TIRADS, thyroid imaging report and data system; US, ultrasonography.

The univariate logistic regression analysis revealed that age, *BRAF V600E* mutation, multifocality, and calcifications in US examination were significantly associated with HVCLNM. Nodule diameter, TIRADS classification, and suspicious malignant focus at the inferior part of thyroid lobe might be associated with HVCLNMs with a *p*-value between 0.05 and 0.10. They were all included in multivariate logistic regression analysis. As shown in **Table S2** and **Supplementary Figure S1**, the risk of HVCLNM in patients with PTC decreased dramatically when the patients were older than 35 years. Thus, age was included in multivariate logistic regression analysis as a dichotomous variable (</=35 years vs. >35 years). Multivariate logistic regression analysis showed that age (</=35 years vs. >35 years), *BRAF* with V600E mutated, nodule diameter, and calcification in US examination independently predicted HVCLNMs (**Table 2**).

**Table 2:** The results of logistic regression analysis.

Variable	Univariate		Multivariate	
	OR (95% CI)	*p*-value	OR (95% CI)	*p*-value
Age (years)		<0.001		<0.001
</=35	1 (reference)		1 (reference)	
>35	0.159 (0.088,0.288)	<0.001	0.095 (0.046,0.193)	<0.001
Gender		0.184		
Female	1 (reference)			
Male	1.566 (0.808, 3.034)	0.184		
BRAF with V600E mutated	4.251 (1.005, 17.988)	0.049	14.363 (2.847, 72.463)	0.001
Hashimoto’s thyroiditis	1.610 (0.890, 2.913)	0.116		
US Characteristics				
Multiple Suspicious Malignant Foci		0.040		
No focus with TIRADS of 4c/5	1 (reference)			
Single focus with TIRADS of 4c/5	2.556 (1.149,5.685)	0.021		
Multiple Foci with TIRADS of 4c/5	1.443 (0.545, 3.825)	0.460		
TIRADS of 4a/4b/4c/5		0.056		
4a	1 (reference)			
4b	3.429 (0.411,28.632)	0.255		
4c	1.957 (0.194,19.700)	0.569		
5	6.136 (0.816, 46.166)	0.078		
Diameter/mm	1.027 (0.997, 1.058)	0.073	1.040 (1.003, 1.079)	0.035
Solid composition	1.092 (0.132, 9.044)	0.935		
A/T > 1	0.754 (0.415, 1.369)	0.353		
Echogenicity		0.133		
Isoechogenicity	1 (reference)			
Hyperechogenicity	0.000^†^	0.999		
Hypoechogenicity	1.840 (0.915, 3.702)	0.087		
Marked hypoechogenicity	0.302 (0.038, 2.433)	0.261		
Margin		0.111		
Well-circumscribed	1 (reference)			
Irregular	1.861 (1.037, 3.340)	0.037		
Microlobulated	1.137 (0.135, 9.567)	0.906		
Calcification		<0.001		<0.001
None	1 (reference)		1 (reference)	
Microcalcification	8.484 (3.278, 21.956)	<0.001	11.221 (4.019, 31.325)	<0.001
Macrocalcification	3.289 (0.964, 11.219)	0.057	4.982 (1.306, 19.006)	0.019
Rim Calcification	4.229 (0.434, 41.215)	0.215	13.044 (1.196, 142.202)	0.035
Vascularity Pattern		0.791		
Absence	1 (reference)			
Perinodular	0.529 (0.154, 1.815)	0.312		
Intranodular	0.991 (0.538, 1.825)	0.976		
Perinodular and intranodular	0.000^†^	0.999		
SMF at inferior part of thyroid lobe	1.952 (0.959, 3.971)	0.065		
SMF at superior part of thyroid lobe	1.386 (0.729, 2.633)	0.319		

†No cases had high-volume central lymph node metastasis in these subgroups.

A/T, the anteroposterior dimension divided by its transverse dimension; SMF, suspicious malignant foci; TIRADS, thyroid imaging report and data system; US, ultrasonography.

**Figure 1:**
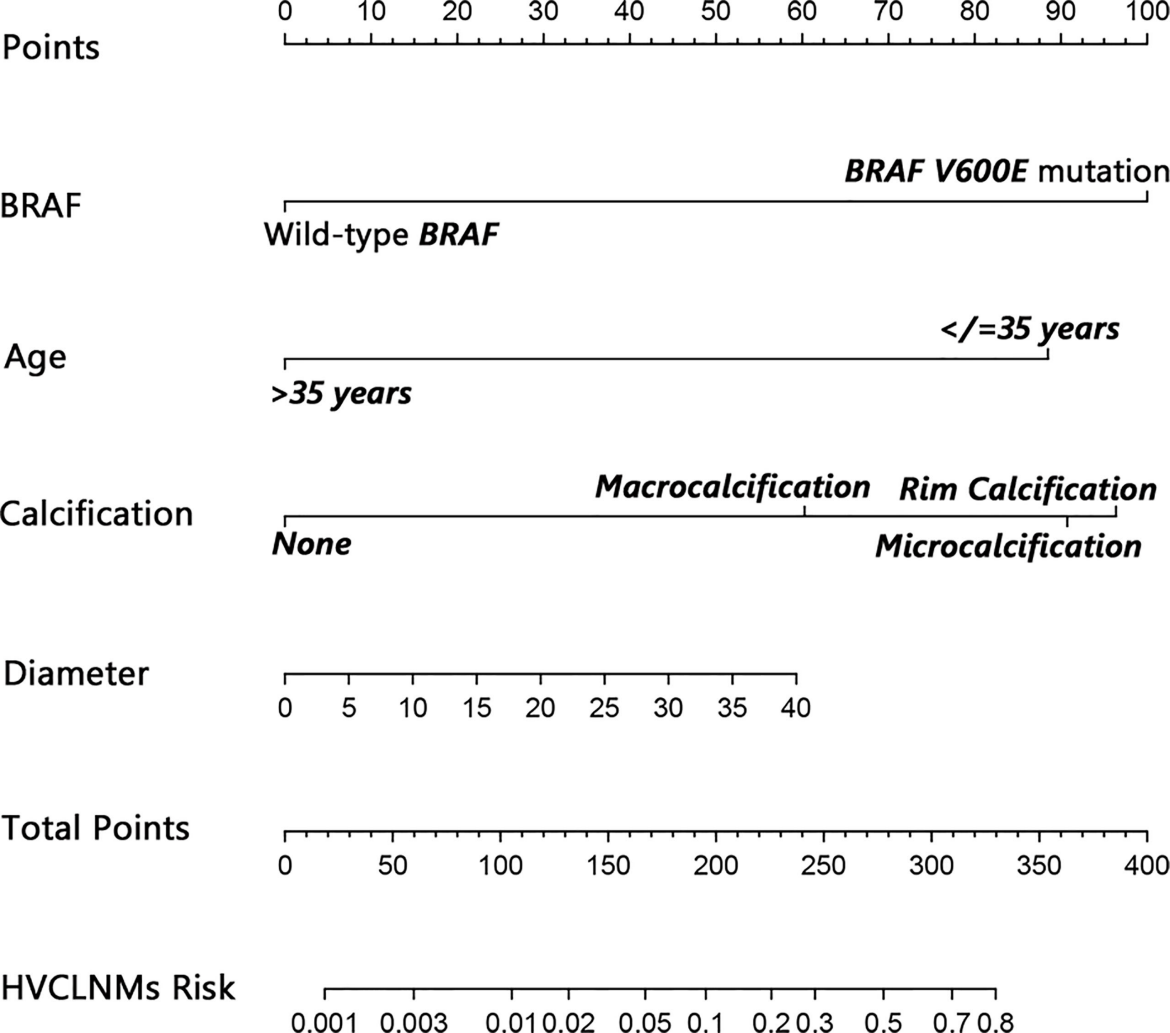
Nomogram to predict high-volume lymph node metastasis in the central compartment (equal to or more than 5 lymph nodes). A 32-year-old (about 87.5 points) patient who has a malignant focus of 20 mm (about 30.0 points) with *BRAF V600E* mutation (100.0 points) and microcalcification (about 90.0 points) would get a total of about 307.5 points, which implies a near 0.70 risk for HVCLNMs. The bottom line indicating the risk of HVCLNMs is given as logarithm. HVCLNMs, high-volume central lymph node metastasis.

**Figure 2:**
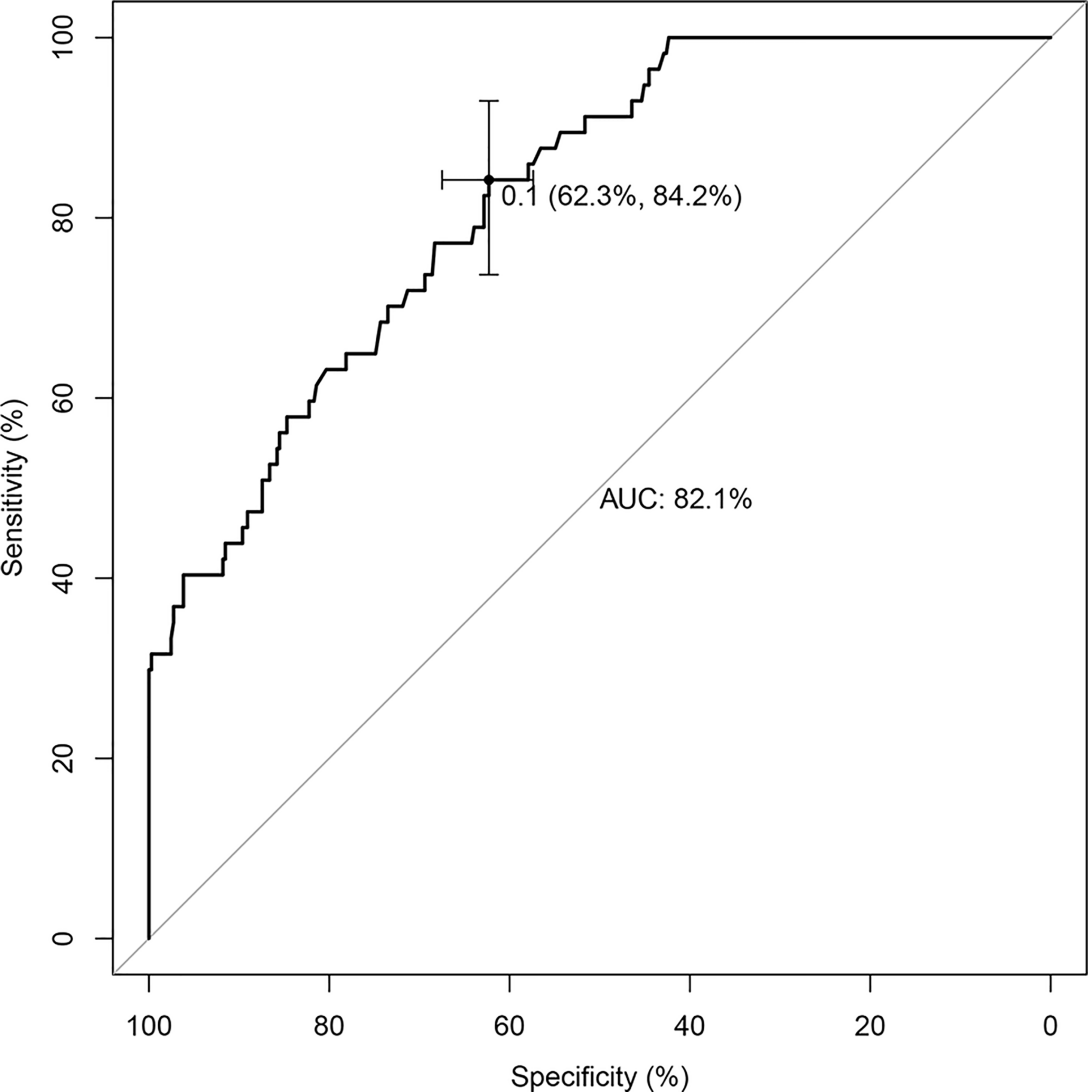
Receiver operator curves of the nomogram.

**Figure 3:**
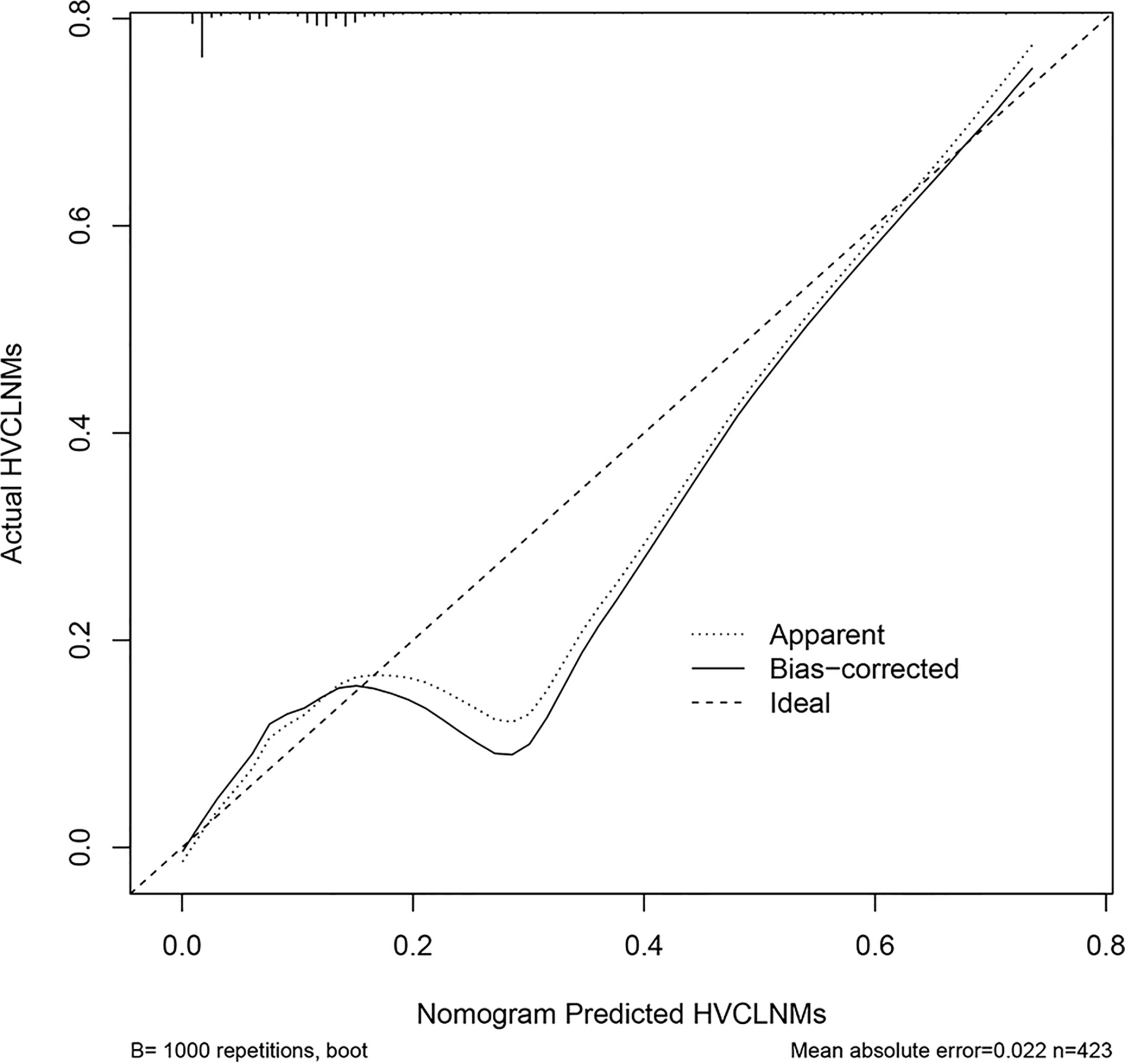
Calibration plot of the nomogram. The *x*-axis is the nomogram-predicted probability of high-volume central compartment lymph node metastasis (HVCLNMs). The *y*-axis is the actual probability of HVCLNMs. Dashed line = ideal nomogram; dotted line = apparent predicted accuracy; solid line = calibration estimate from the internally validated model. Perfect prediction would correspond to the dashed line.

The nomogram that incorporated the independently predictive factors was developed and presented (**Figure 1**). The mean AUC for the nomogram was 0.821 (95% CI, 0.768–0.875) (**Figure 2**). Setting a cutoff point of 0.50, sensitivity, specificity, positive predictive value, and negative predictive value were 40.4%, 96.2%, 62.2%, and 91.2%, respectively. The calibration curve of the nomogram presented good agreement between the predicted and observed probability of HVCLNMs, especially for the observed HVCLNMs probability >0.5 (**Figure 3**).

## Discussion

In the recent two decades, several studies indicated that oncologic outcomes of lobectomy are comparable to those of a near-total or total thyroidectomy for low-risk papillary thyroid carcinoma. Davies et al. analyzed 35,223 cases of PTC confined to the thyroid gland from 1973 to 2005 and found that the prognosis of the patients with either hemithyroidectomy or total thyroidectomy would be the same (1). Bilimoria et al. analyzed 52,173 patients having underwent surgery for PTC from 1985 to 1998 and concluded that extent of surgery (lobectomy versus total thyroidectomy) did not impact recurrence or survival for PTC <1 cm (2). Jonklaas et al. drew a similar conclusion that the extent of thyroid surgery was not associated with overall survival, disease-specific survival, or disease-free survival in stage I patients (3). In the 2015 ATA guidelines, thyroid lobectomy alone was recommended as an alternative for the initial treatment of low-risk PTC (8). A similar opinion was presented in NCCN clinical practice guidelines in oncology (thyroid carcinoma, version 3.2021) (7). However, on account of the unsatisfying diagnostic performance of imaging techniques, some adverse features, which indicate high risk of recurrence and reveal unfavorable prognosis, could only be assessed in postoperative pathological examination. Completion thyroidectomy following lobectomy is recommended to reduce risk of recurrence and improve prognosis for these patients, whereas completion thyroidectomy following lobectomy may lead to increasing complications, severe anxiety of the patients, and extra expenditures for medical care and public health, compared with one-stage surgery (near-total or total thyroidectomy) (9, 20).

HVLNMs (equal to or more than 5 involved lymph nodes) was one of the risk factors indicating high recurrence rate and poor prognosis in several studies (21). Sugitani et al. reported that the patients with > 5 LN metastases demonstrated a significantly higher risk of recurrence (19% versus 8%) (11). Likewise, in the research by Leboulleux et al., the patients with >10 positive LNs (21%) or 6–10 LN metastases (7%) significantly predicted higher 10-year risk of recurrence compared with the patients with <5 LN metastases (3%) (10). In the 2015 ATA guidelines, pathologic N1 with more than 5 involved lymph nodes was one of the criteria to upgrade low-risk PTC to intermediate-risk PTC, which makes risk of structural disease recurrence increase by about 15% (8). Furthermore, HVLNMs is recommended as one of the adverse pathological features indicating completion thyroidectomy following lobectomy in NCCN clinical practice guidelines in oncology (thyroid carcinoma, version 3.2021) (7). However, the diagnostic performance of preoperative US or computer tomography in predicting lymph node metastasis (LNM) in the central compartment is unsatisfactory for now, which is the most frequently involved level in PTC (8, 12, 22). Thus, it may be valuable to develop a predictive model in evaluating the status of lymph nodes and predicting HVLNM in the central compartment, which may help surgeons and patients draw up individual treatment plans preoperatively and reduce reoperation risk. In our research, we developed a nomogram based on preoperative clinical features, in which the AUC was 0.821 (95% CI, 0.768–0.875) in predicting HVCLNMs. These results validate the idea that our nomogram provides good discrimination ability for detecting HVCLNMs in PTC patients (23, 24). Furthermore, the nomogram is easy to use, repeated, and economical, especially in the undeveloped area. In the 2015 ATA guidelines, lateral neck compartmental lymph node dissection was recommended for the patients with biopsy-proven metastatic lateral cervical lymphadenopathy (8). Thus, in this cohort, lateral neck dissection was not performed for the patients. During the follow-up period, the lateral neck lymph node should be actively surveilled for the patients with HVCLNMs. If lateral neck lymph node recurrence increases significantly in patients with HVCLNMs, elective neck dissection should be considered in these cases.

V-raf murine sarcoma viral oncogene homolog B1 (*BRAF*) mutations play roles in tumor cell proliferation and differentiation (25). *BRAF V600E* is the most common oncogene in PTC, which is reported to be correlated with some clinicopathological features in some studies (26, 27). In the research by Shi et al., a cohort of 126 patients was retrospectively analyzed and *BRAF* mutation status was found to be significantly associated with tumor size and LNM (28). A meta-analysis, including 3,437 patients, showed that the *BRAF V600E* mutation was correlated with some aggressive clinicopathological features, such as tumor multifocality, extrathyroidal extension, lymph node metastases, and advanced stage of PTMC (29). Similarly, Chen et al. reported that *BRAF (V600E)* mutation was an independent risk factor for central lymph node metastasis (CLNM), together with size </=10mm, microcalcification, internal vascularity, and capsule contact or involvement in preoperative ultrasound examination (30). Although several studies reported the association between *BRAF (V600E)* mutation and CLNM, no study focused on the predictive value of *BRAF (V600E)* mutation for HVCLNMs and the assistance in clinical decision-making of thyroidectomy. In our research, positive *BRAF V600E* mutation in PTC was proved to be able to predict high-volume CLNM independently with an odds ratio of 14.363 (95% CI, 2.847–72.463), which indicated that the preoperative *BRAF V600E* mutation test is useful in risk stratification of HVCLNMs and could assist in the management of PTC.

Age was reported to be associated with LNM in several studies previously (21, 31–35). Liu et al. included a total of 48,166 PTC and concluded that LNM rate decreased with age, especially women (*p* < 0.0001) (31). Luo et al. included 1,031 patients with PTMC and reported that male, age (</= 40 years), tumor largest diameter (>/= 5 mm), multifocal, non-uniform echoic distribution, the sum of the maximum diameter of multifocal in a unilateral lobe (>/= 8.5 mm), and tumors in the lower pole location were risk factors of CLNM in papillary thyroid microcarcinoma (PTMC) (32). Another study about PTMC similarly reported that young (<40 years old) and male patients were independent risk factors for large‐volume LNM (>5 metastatic lymph nodes) (21). In our study, the analysis drew a similar conclusion that young age (</=35 years) was an independent risk factor for high-volume CLNM, which could effectively predict HVCLNMs, together with *BRAF V600E* mutation, nodule diameter, and nodule calcification in US.

Our study has several limitations. Firstly, in our study, we only focused on high-volume CLNM. For the patients with high risk of HVCLNMs, total thyroidectomy should be recommended more strongly. Otherwise, the risk of other adverse features, such as macroscopic multifocal disease and vascular invasion, should be assessed at the same time, which would assist in determining surgical procedure, total thyroidectomy versus thyroid lobectomy, for the patients with low risk of HVCLNMs. Secondly, the nomogram was assessed only using the internal validation method, and this might have some adverse impact on the application in other centers. In the near future, the diagnostic performance of the nomogram may be further improved *via* multicentral research with a large sample.

## Conclusion

The preoperative nomogram performed well in discriminating the patients with or without high-volume CLNM (equal to or more than 5 lymph nodes) in papillary thyroid carcinoma, which is one of the indications for completion thyroidectomy after thyroid lobectomy.

## Data Availability Statement

The datasets used during the current study are available in Mendeley Data, V1, doi: 10.17632/sd59nt5dty.1.

## Ethics Statement

The studies involving human participants were reviewed and approved by the Ethics Committee of Sun Yat-sen Memorial Hospital, Sun Yat-sen University. Written informed consent to participate in this study was provided by the participants’ legal guardian/next of kin.

## Author Contributions

PL: Conceptualization, Methodology, Software, Formal analysis, Investigation, Data Curation, and Writing—Original Draft. FL: Methodology, Formal analysis, Investigation, Resources, and Writing—Original Draft. JR: Conceptualization, Formal analysis, Investigation, Resources, Data Curation, and Writing—Original Draft. PH: Validation, Investigation, Writing—Original Draft, and Funding acquisition. JL: Conceptualization, Validation, Investigation, Resources, Writing—Original Draft, and Funding acquisition. RC: Validation, Formal analysis, Investigation, Writing—Original Draft, and Visualization. BL: Methodology, Resources, Writing—Review and Editing, Supervision, and Project administration. NO: Conceptualization, Methodology, Writing—Review and Editing, Supervision, and Project administration. XH: Conceptualization, Methodology, Resources, Data Curation, Writing—Review and Editing, Supervision, and Funding acquisition. All authors contributed to the article and approved the submitted version.

## Funding

This study was supported by the Grants from the Sun Yat-Sen University Clinical Research 5010 Program (Grant 2010008), the National Natural Science Foundation of China (Nos. 81872193, 81702697, and 81903043), and the Natural Science Foundation of Guangdong Province (No. 2018A030310086). The sponsors were not involved in research activities, such as study design, analysis, and interpretation of data.

## Conflict of Interest

The authors declare that the research was conducted in the absence of any commercial or financial relationships that could be construed as a potential conflict of interest.

## Publisher’s Note

All claims expressed in this article are solely those of the authors and do not necessarily represent those of their affiliated organizations, or those of the publisher, the editors and the reviewers. Any product that may be evaluated in this article, or claim that may be made by its manufacturer, is not guaranteed or endorsed by the publisher.

## Supplementary Material

Supplementary Figure S1 Correlation analysis of age at diagnosis and HVCLNMs rate in patients with papillary thyroid carcinoma. Error bars: 95% confidence interval. HVCLNMs, high-volume central lymph node metastasis.

## Abbreviations

A/T, the anteroposterior dimension divided by its transverse dimension; AUC, area under receiver operator characteristic curve; CI, confidence interval; CLNM, central lymph node metastasis; FNAC, fine-needle aspiration cytologic examination; HVCLNMs, high-volume central lymph node metastasis (equal to or more than 5 involved lymph nodes) in central compartment; HVLNMs, high-volume lymph node metastasis (equal to or more than 5 lymph nodes); KWAK-TIRADS, thyroid imaging report and data system developed by Kwak et al.; LN, lymph node; LNM, lymph node metastasis; LOD, limit of detection; PTC, papillary thyroid carcinoma; PTMC, papillary thyroid microcarcinoma; ROC, receiver operator characteristic curve; TgAb, thyroglobulin antibody; TIRADS, thyroid imaging report and data system; TPOAb, thyroperoxidase antibody; US, ultrasonography.
