# Supplementary figures and images for: A Preoperative Nomogram for the Prediction of High-Volume Central Lymph Node Metastasis in Papillary Thyroid Carcinoma
Source: Front Endocrinol (Lausanne). 2021 Dec 22;12:753678. doi: 10.3389/fendo.2021.753678 (PMC8729159; doi:10.3389/fendo.2021.753678)

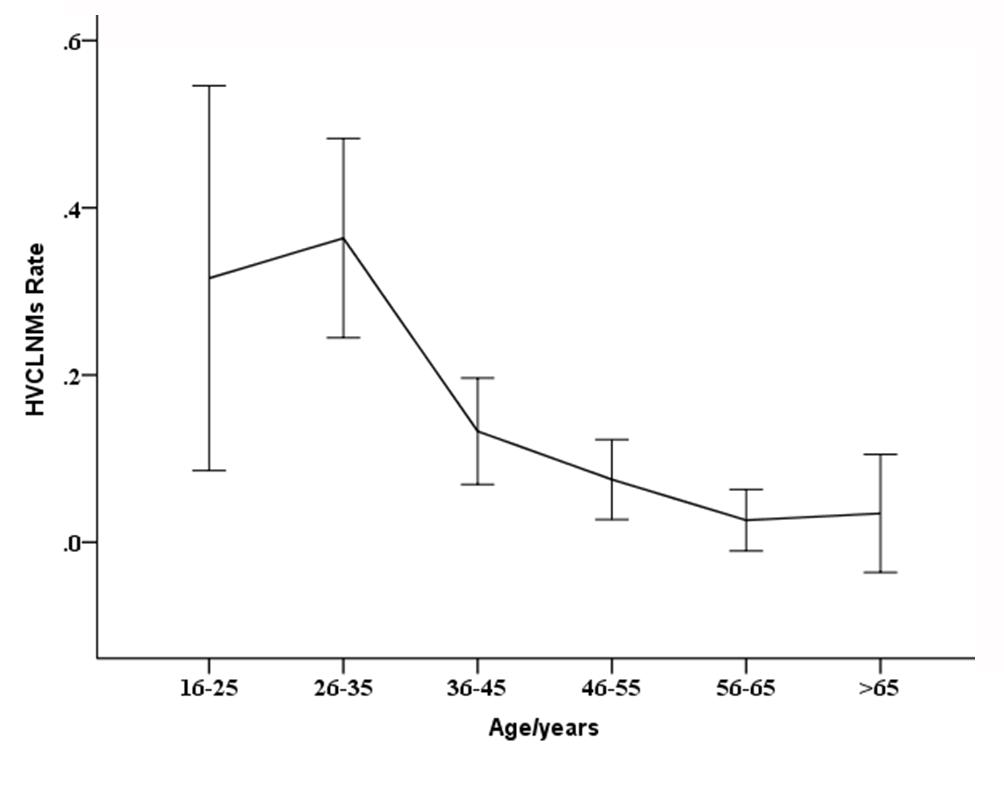

Supplement: Supplementary Figure S1 — Correlation analysis of age at diagnosis and HVCLNMs rate in patients with papillary thyroid carcinoma. Error bars: 95% confidence interval. HVCLNMs, high-volume central lymph node metastasis. [file Image_1.tif]
