# Supplementary material for: A Preoperative Nomogram for the Prediction of High-Volume Central Lymph Node Metastasis in Papillary Thyroid Carcinoma
Source: Front Endocrinol (Lausanne). 2021 Dec 22;12:753678. doi: 10.3389/fendo.2021.753678 (PMC8729159; doi:10.3389/fendo.2021.753678)
Supplement: Supplementary file 2 [file DataSheet_1.docx]

Table S1 The intra- and inter-observer agreements in assessing US characteristics

| Kappa coefficient | Intra-observer agreement | | Inter-observer agreement |
| --- | --- | --- | --- |
|  | Dr.L | Dr.R |  |
|  | n=100 | n=100 | n=423 |
| Composition | 0.773±0.110 | 0.839±0.092 | 0.796±0.115 |
| Shape | 0.945±0.016 | 0.955±0.015 | 0.900±0.022 |
| Echogenicity | 0.964±0.013 | 0.959±0.014 | 0.930±0.018 |
| Margin | 0.951±0.015 | 0.960±0.013 | 0.946±0.015 |
| Calcification | 0.966±0.011 | 0.977±0.009 | 0.947±0.014 |
| Vascularity Pattern | 0.932±0.017 | 0.957±0.013 | 0.931±0.017 |

US, ultrasound.

Table S2 Different HVCLNMs rate in young and old patients

| Age/years | HVCLNMs/cases | | HVCLNMs rate/% |
| --- | --- | --- | --- |
|  | No | Yes |  |
| </=35 | 55 | 30 | 35.3 |
| >35 | 311 | 27 | 8.0 |

HVCLNMs, high-volume central lymph node metastasis (equal to or more than 5 lymph nodes).
